# Integrative bulk and single-cell transcriptomics link EZH2 to immunosuppressive programs and tumor–Treg crosstalk in castration-resistant prostate cancer

**DOI:** 10.3389/fimmu.2026.1725097

**Published:** 2026-02-03

**Authors:** Xing Xiong, Jianhu Xie, Ping Dai, Chao Cheng, Jiani Liu, Guixiao Huang

**Affiliations:** 1 Department of Urology, The Third Affiliated Hospital of Shenzhen University, Shenzhen, China; 2 Department of Data Analysis, Biosalt Inc, Wuhan, China

**Keywords:** CRPC, EZH2, immunosuppressive, prostate cancer, single-cell RNA-seq, Treg

## Abstract

**Background:**

Enhancer of zeste homolog 2 (EZH2) is frequently upregulated in prostate cancer (PCa) and further increased in castration-resistant prostate cancer (CRPC), a lethal state characterized by profound immune dysfunction. However, EZH2-associated immune programs in bulk cohorts and their corresponding cell-type–specific features in single-cell RNA sequencing (scRNA-seq) data have not been systematically delineated in advanced PCa.

**Methods:**

We integrated bulk RNA-seq and scRNA-seq to map EZH2-associated transcriptional and immune features in PCa. In bulk cohorts (TCGA-PRAD and an independent metastatic CRPC cohort), we quantified EZH2 expression, clinical outcomes, and immune-signature enrichment. Immune-modulated differentially expressed genes (IMDEGs) were defined by intersecting EZH2-associated differential expression with correlations to Treg/TAM-related signature scores, and were used for NMF-based immune subtyping and penalized Cox modeling with validation. In scRNA-seq cohorts (GSE264573 and an independent CRPC cohort), malignant epithelial cells were inferred by copy-number alteration profiles, EZH2^high versus EZH2^low malignant programs were characterized, T-cell subsets were quantified, and tumor–Treg communication was inferred using CellPhoneDB as hypothesis-generating predictions. For perturbation, the EZH2 inhibitor tazemetostat was evaluated in the CRPC-relevant C42 cell line with H3K27me3 readouts and transcriptomic profiling, with key changes validated by RT–qPCR.

**Results:**

Across bulk cohorts, higher EZH2 expression was associated with adverse clinical outcomes and increased enrichment of immunosuppressive signatures, including Treg- and TAM-related programs. IMDEG-based NMF subtyping identified patient groups with distinct immune states, and an IMDEG-derived risk score stratified prognosis. Single-cell profiling revealed elevated EZH2 in CRPC malignant cells and Tregs; EZH2^high malignant cells exhibited a proliferative transcriptional state accompanied by reduced expression of immune-related programs. Predicted tumor–Treg interaction patterns were stronger in CRPC and positively associated with EZH2 expression. In C42 cells, tazemetostat reduced H3K27me3 and induced coordinated transcriptional changes, including upregulation of immune- and inflammation-associated genes such as TIMP3, PLCG2, and SOCS3, validated by RT–qPCR.

**Conclusion:**

This multi-layer integrative analysis suggests that EZH2 is associated with proliferative malignant states and immunosuppressive microenvironment features in advanced PCa, including Treg-linked crosstalk. Transcriptomic profiling following EZH2 inhibition supports modulation of these programs by EZH2-targeted perturbation, while functional and causal mechanisms warrant further investigation.

## Introduction

Prostate cancer (PCa) is the second most commonly diagnosed cancer in men worldwide, with ~1.47 million new cases in 2022 (≈14.2% of all male cancers) (1). While early-stage disease is often manageable, advanced cases present significant therapeutic challenges (2). Androgen deprivation therapy (ADT) is initially effective in controlling advanced PCa; however, most patients eventually progress to a castration-resistant state (CRPC) despite hormone suppression (3, 4). CRPC represents a lethal stage with limited treatment options and poor prognosis. Compounding this challenge is the fact that prostate tumors are relatively immunologically cold. CRPC typically exhibits low levels of T-cell infiltration and a low neoantigen burden, along with an abundance of immunosuppressive factors in the tumor microenvironment (TME) (5). As a result, immune checkpoint inhibitors (ICIs) such as anti-PD-1 or anti-CTLA-4 have shown minimal efficacy in PCa, in stark contrast to the dramatic immunotherapy responses seen in more immunogenic tumors (6, 7). The CRPC TME is often dominated by suppressive cell populations—notably regulatory T cells (Tregs), myeloid-derived suppressor cells, and M2-polarized tumor-associated macrophages (TAMs)—which create formidable barriers to effective anti-tumor immunity (8). These features underscore the need for strategies that can reverse immune evasion in the PCa tumor microenvironment, potentially by targeting epigenetic repressors such as EZH2 that remodel tumor transcriptional programs relevant to tumor–immune interactions.

Enhancer of zeste homolog 2 (EZH2), the catalytic subunit of the polycomb repressive complex 2 (PRC2), exerts its biological functions by catalyzing the trimethylation of histone H3 on lysine 27 (H3K27me3)—an epigenetic modification tightly linked to gene silencing (9). Its dysregulated overexpression represents a prominent hallmark of multiple solid tumors, correlating with enhanced cancer cell proliferation and profound remodeling of the tumor immune landscape (10). EZH2 is highly expressed in tumor-infiltrating regulatory T cells (TI-Tregs), where it maintains the stability of FOXP3 and reinforces the immunosuppressive function of TI-Tregs, thereby suppressing anti-tumor immunity (11, 12). As the disease progresses to advanced stages, EZH2 expression is progressively upregulated, which acts as a key driver of PCa initiation and progression (13) and serves as a marker of poor prognosis (14, 15). Correspondingly, EZH2-hyperactive PCa tumors typically exhibit an immunosuppressive TME and intrinsic resistance to immunotherapy (13). Mechanistically, EZH2 mediates PCa immune escape primarily through multifaceted epigenetic regulation. On the one hand, it silences interferon-stimulated genes (ISGs), Th1 chemokines, MHC-I-related genes and endogenous retroviral sequences via H3K27me3 modification, which impairs T cell recognition, blocks the activation of the STING pathway in innate immunity, and modulates the polarization of TAMs to maintain an immunosuppressive TME and induce resistance to PD-1 inhibitors—an effect that can only be abrogated by combination treatment with PD-1 blockers (13); on the other hand, EZH2 epigenetically represses immune checkpoint molecules and other immune-related genes, regulates the differentiation of CD4^+^ T cells and Tregs, and inhibits the infiltration of CD8^+^ cytotoxic T cells, thereby further shaping an immunosuppressive TME and enhancing resistance to immunotherapy (16). However, how EZH2 synergistically regulates the crosstalk between malignant cells and immune cells (e.g., Tregs, TAMs) to drive CRPC immune evasion, as well as the key downstream molecules and signaling axes involved, remain to be systematically elucidated.

Given EZH2’s dual roles in tumor progression and immune escape, this study aimed to define how EZH2 coordinates immunosuppression in CRPC. We integrated bulk RNA-seq and single-cell RNA-seq (GSE264573 and an independent CRPC scRNA-seq cohort, GSE137829) data; quantified associations between EZH2 expression and immune signature enrichment; derived an EZH2-stratified set of immune-modulated differentially expressed genes (IMDEGs) with a risk model and non-negative matrix factorization (NMF)-based immune subtypes; and assessed EZH2-linked programs and malignant cell–Treg interactions at single-cell resolution. Finally, in C42 cells, we profiled the transcriptional effects of EZH2 inhibition, confirming target engagement by reduced H3K27me3 and identifying coordinated drug-responsive transcriptional programs. Together, these analyses indicate that higher EZH2 expression coincides with an immunosuppressive microenvironment in CRPC—characterized by elevated Treg signature enrichment (validated by single-cell quantification of Treg proportions), reduced antigen-presentation signals, and strengthened malignant cell–Treg communication—and nominate EZH2-related networks as testable targets for immunomodulatory strategies in PCa.

## Materials and methods

### Bulk transcriptome datasets

#### Data acquisition and preprocessing

Bulk RNA-seq data were compiled from TCGA-Prostate Adenocarcinoma (PRAD) and the SU2C/PCF Dream Team 2019 mCRPC study (SU2C/PCF 2019) (17). For TCGA, the raw counts matrix, gene-level FPKM expression matrix and phenotype were downloaded from UCSC Xena (accessed on 2025/7/12; Xena cohort/hub: GDC) (18). FPKM values were converted to TPM column-wise rescaling (TPM = FPKM/ΣFPKM × 10^6 for each sample). We retained primary tumor samples and mapped Ensembl IDs to HGNC symbols using Gencode v22; when multiple Ensembl IDs mapped to the same symbol, the entry with the highest mean expression across samples was retained.

For SU2C/PCF 2019, RNA-seq expression and sample annotations were obtained from cBioPortal (accessed on 2025/8/13; study ID: prad_su2c_2019). We used the study-provided mRNA expression matrix (FPKM) and applied the same TPM transformation. Where multiple biopsies existed for the same patient, we de-duplicated to one representative sample per PATIENT_ID (selection rule: the first available biopsy) to avoid pseudo-replication and over-weighting individuals in downstream association and survival analyses. For these two datasets, if multiple rows mapped to the same symbol, the row with the highest mean expression was kept.

Unless otherwise stated, bulk analyses using single-sample GSEA (ssGSEA) and correlation analyses were performed on TPM expression values, whereas differential expression by DESeq2 used raw counts. A complete list of all datasets used in this study, together with sample size and key clinical/technical metadata, is provided in **Supplementary Table 1**. Clinical variables for TCGA-PRAD and the SU2C/PCF 2019 cohort are summarized in **Supplementary Table 2**.

#### Survival analyses

Survival was evaluated as PFI in TCGA-PRAD and OS in SU2C/PCF 2019. Kaplan–Meier curves were generated and compared using log-rank tests, and Cox proportional hazards models were fitted using the survival package in R (v4.4.3). For visualization (Kaplan–Meier displays), patients were dichotomized at the cohort median of the indicated variable. Time-to-event was analyzed in days, and individuals without events were censored at last follow-up.

#### Immune/stromal signature scoring

We obtained a set of 29 predefined functional gene-expression signatures from a prior pan-cancer immune-deconvolution resource (19), covering anti-tumor immunity, pro-tumor immunosuppression, stromal/angiogenic activity, and malignant-cell hallmarks (**Supplementary Table 3**). For each cohort, we computed per-sample enrichment scores using ssGSEA implemented in GSVA (v1.32.0) (20) in R; scores were mean-centered and z-scaled within cohort for visualization and correlation analyses. Associations with EZH2 were assessed using Spearman rank correlation; and only features with P-value < 0.05 are displayed.

#### IMDEG construction

To derive EZH2-associated immune-modulated differentially expressed genes (IMDEGs), we first performed bulk differential expression (DE) in TCGA-PRAD by contrasting EZH2-high (top 30 samples) versus EZH2-low (bottom 30 samples) tumors using DESeq2 (v1.42.0) (21); genes with BH FDR < 0.05 and |log2FC| ≥ 1 were considered significant, and functional enrichment was computed on the DE set using clusterProfiler package (v4.6.2) (22). Next, we quantified Treg and TAM/M2 signatures per sample (ssGSEA/GSVA; see above) and computed Spearman correlations between each gene’s expression and these signature scores. IMDEGs were defined as EZH2-associated DE genes that also showed significant association with Treg or TAM/M2 (|r| ≥ 0.4, P-value < 0.05) using a moderate-effect correlation cutoff to prioritize robust, biologically meaningful associations while limiting weak, potentially spurious correlations. Enrichment of IMDEG terms was re-assessed with clusterProfiler.

#### Machine-learning survival modeling and validation

A parsimonious prognostic model was developed using a Cox proportional hazards model with L1-penalization (LASSO) to minimize overfitting (glmnet v4.1.8). All model development steps were restricted to the training cohort to prevent information leakage. Gene expression values were log-transformed (log1p) and then standardized using z-scores derived only from the training cohort; the same training-derived centering and scaling parameters were applied unchanged when computing predictors in the external cohort.

Model tuning was performed by cross-validation within the training cohort (5-fold CV; λ selected by lambda_1se) to select the regularization strength (λ). The final model yielded a continuous risk score for each patient, calculated as the weighted sum of standardized gene expression values using the LASSO-derived coefficients. Discrimination was evaluated using Harrell’s concordance index (C-index) and time-dependent ROC curves (timeROC v0.4; time points: 1/3/5 years) in the training cohort.

For external validation, the fixed risk-score formula learned in the training cohort (including coefficients and the training-derived standardization parameters) was directly applied to the independent SU2C/PCF 2019 prostate cancer cohort. External discrimination was quantified using C-index and time-dependent ROC. Because available endpoints differ across cohorts (PFI in TCGA-PRAD and OS in SU2C/PCF 2019), validation focused on risk stratification and discrimination consistency under each cohort’s recorded endpoint. To assess whether the risk score provides prognostic information beyond clinicopathologic variables, multivariable Cox models including the risk score and clinical covariates (Gleason score, pathological T stage, and PSA) were fitted, and hazard ratios (HRs), 95% confidence intervals (CIs), and p-values were reported.

#### NMF subtyping and immune-modulator profiling

We applied NMF (NMF v0.28) to the DESeq2 variance-stabilized (VST) expression matrix restricted to IMDEGs (genes × samples) with ranks k = 2–10, 50 runs per k, and fixed random seeds (algorithm = brunet). The optimal k was selected using the cophenetic correlation coefficient, dispersion, and silhouette width; samples were assigned to IMDEG-based immune subtypes by maximum basis coefficient/consensus membership. After subtype assignment, we profiled a curated panel of 142 immune regulators from Hu et al. based on the pan-cancer framework of Charoentong et al. (42 stimulators, 23 inhibitors, 39 chemokines, 18 chemokine receptors, 20 MHC genes) (23, 24) (gene list provided in **Supplementary Table 5**). For visualization, immune-modulator and IMDEG expression values were row-wise z-scaled and displayed as heatmaps ordered by NMF subtype with category annotations.

### Single-cell RNA-seq

#### Data source and quality control

The scRNA-seq dataset GSE264573 (CSPC and CRPC tumors) (25) was downloaded as gene-barcodes matrices and analyzed with Seurat (v5.1.0) (26). Cells were filtered by standard QC thresholds: nFeature_RNA 300–8,000, nCount_RNA > 500, mitochondrial percentage < 20%. Cells with ≥10% of total UMIs mapping to hemoglobin genes (HBA1, HBA2, HBB, HBD, HBG1/2, ALAS2) were flagged as erythrocytes and excluded from downstream analyses. Genes expressed in < 3 cells were excluded. After QC, a total of 94,356 cells from 13 CSPC and 11 CRPC samples were retained for downstream analyses (44,428 CSPC cells and 49,928 CRPC cells). No explicit doublet-removal algorithm was applied beyond the above QC filters; residual doublets, if present, are expected to have limited impact on the main conclusions based on broad cell-state patterns and marker-supported annotations.

To externally validate the malignant cell–intrinsic EZH2-associated programs, we analyzed an independent human CRPC scRNA-seq cohort (GSE137829; 6 CRPC samples) using the same preprocessing and annotation framework as in the discovery cohort. This validation cohort was used specifically to assess the reproducibility of malignant cell–intrinsic EZH2-associated transcriptional programs.

#### Normalization, integration, and clustering

Raw counts were normalized with Seurat’s LogNormalize and highly variable genes were identified (vst, n_features = 2000). To correct batch effects across patients and disease states (CSPC, CRPC), we applied Seurat’s CCA-based integration: SelectIntegrationFeatures, FindIntegrationAnchors (reduction = ‘cca’, dims = 1:50), and IntegrateData to obtain an integrated expression matrix. The integrated assay was then scaled and subjected to PCA (50 components), followed by neighbor graph construction (FindNeighbors using dims = 1:50), UMAP embedding, and clustering (FindClusters, resolution = 0.6). Lineages were annotated by canonical markers and cross-checked with ScType (27): Epithelial (EPCAM/KRT8/18), Fibroblasts (COL1A1/DCN/LUM), Endothelial (PECAM1/VWF), T cells (CD3D/CD3E), B cells (CD79A/MS4A1), Monocytes/Macrophages (LYZ/CD68), Mast (CPA3/TPSAB1).

#### Malignant cell calling by CNA inference

Copy-number alterations (CNA) were inferred from scRNA-seq using inferCNV (v1.22.0) (https://github.com/broadinstitute/inferCNV) with gene ordering from GENCODE v38 (GRCh38/hg38) GTF. B cells served as reference. inferCNV was run following the recommended workflow (default parameters unless otherwise specified). Two per-cell metrics were derived: CNA signal (amplitude) and CNA correlation with the malignant reference profile. Cutoffs were set at the 90^th^ percentile of reference cells for CNA signal and 80^th^ percentile for CNA correlation to control false-positive malignant calls by anchoring thresholds to the upper tail of the non-malignant reference distribution, while retaining sensitivity for high-CNA epithelial populations. Cells exceeding both cutoffs were labeled malignant; those exceeding one cutoff unresolved; and those below both non-malignant. Results were visualized as heat maps and scatterplots.

#### Differential expression and pathway analysis in malignant cells

Malignant cells were stratified by EZH2 expression (top 25% vs bottom 25% within malignant cells). DE between EZH2^high and EZH2^low malignant cells used Seurat’s Wilcoxon rank-sum test with min.pct = 0.1, logfc.threshold = 0.25 and BH FDR < 0.05. Hallmark GSEA was performed on logFC-ranked genes using gseapy (28) (MSigDB Hallmark gene sets; access date: 2025/8/15).

#### T-cell subclustering and composition analysis

T cells (PTPRC^+, CD3E^+) were subsetted, re-integrated and reclustered (using the same normalization and integration settings as above; dims = 25, resolution = 0.6). Subsets were annotated by markers: CD4_Tcm (CCR7/IL7R/CD4), CD8_Cytotoxic (CD8A/GZMB/PRF1/GNLY), CD8_Exhausted (CD8A/LAG3/PDCD1/TOX/IFNG), Treg (FOXP3/IL2RA/CTLA4/TIGIT), Cycling_T (MKI67/TOP2A/BIRC5), NK (KLRD1/KLRF1/GNLY/FCGR3A), γδ T (TRDC/XCL1/XCL2). EZH2 and FOXP3 expression distributions in Tregs were summarized by dot plots.

#### Ligand–receptor interaction analysis

Cell–cell communication was inferred using CellPhoneDB (v5) (29) on malignant and Treg clusters from the integrated scRNA-seq dataset combining CSPC and CRPC samples; the filtered cluster-level expression matrix was used as input. Raw counts with gene symbols were used; only genes expressed in ≥10% cells of a cluster were retained. Statistical significance was evaluated by 1,000 permutations. Interactions were filtered by mean interaction score > 2 and permutation p < 0.05, focusing on pairs highlighted in the main text.

#### Metacell-based correlation analysis between EZH2 and other genes

To reduce sparsity and avoid cell-level pseudoreplication, we constructed metacells using hdWGCNA (v0.4.5; gene_select = “fraction”, fraction = 0.05) and MetacellsByGroups (group.by = c(“seurat_clusters”, “group”), k = 25, reduction = “pca”, max_shared = 10), followed by metacell normalization (NormalizeMetacells). The resulting log-normalized metacell expression matrix (RNA assay “data” slot) was used to compute Pearson correlations (two-sided cor.test) between EZH2 and selected genes across metacells. Associations were considered significant at P-value < 0.05.

### Cell culture, EZH2 inhibition, RNA-seq, and RT-qPCR

#### Cell lines and drug treatment

C42 cells were maintained in RPMI-1640 supplemented with 10% fetal bovine serum and 1% penicillin–streptomycin at 37°C in 5% CO_2_. Cells were treated with the EZH2 inhibitor tazemetostat (MedChemExpress, HY-13803) dissolved in DMSO; vehicle controls received an equivalent volume of DMSO. The working condition (10 µM for 24 h) was selected based on a pilot dose titration (0.1–10 µM) assessing pharmacodynamic target engagement by immunoblotting of H3K27me3 with normalization to total H3 (H3K27me3/H3), where 10 µM produced the maximal reduction in the ratio under the same exposure window (antibodies: H3K27me3 [CST, 9733T], total H3 [Proteintech, 68345]; densitometry performed using ImageJ v1.54g). Cell viability under the selected condition was evaluated by Cell Counting Kit-8 (CCK-8) assay prior to transcriptomic profiling to confirm non-cytotoxic exposure.

#### RNA extraction, library preparation, and sequencing

Total RNA was extracted with a silica-column kit (Vazyme, RC112-01) including on-column DNase I digestion. RNA integrity was assessed on an Agilent 2100 Bioanalyzer, and samples with RIN ≥ 8.0 were advanced. Poly(A)+ libraries (Illumina Stranded mRNA Prep; per manufacturer’s instructions) were prepared and sequenced on an Illumina NovaSeq 6000 to yield ~30–50 million paired-end 150-bp reads per sample. Three biological replicates were generated per condition.

#### RNA-seq processing and DEG calling (C42 ± EZH2i)

Reads were quality-checked FastQC (v0.11.9), adapter-trimmed with fastp (v0.23.4) (30), and aligned to GRCh38 with STAR (v2.7.10b; two-pass mode) (31). Gene-level counts were obtained with featureCounts (Subread 2.0.3) (32). DEGs (EZH2i vs control) were identified by DESeq2 with P-value < 0.05 and |log2FC| ≥ 0.585 (corresponding to ≥1.5-fold change).

#### RT-qPCR validation

cDNA was synthesized using a reverse transcription kit (Vazyme, R433-01). qPCR was performed with SYBR Green master mix (Vazyme, Q226-01) on a real-time PCR platform using primer pairs targeting TIMP3, PLCG2, SOCS3, and the housekeeping gene (GAPDH); sequences are provided in **Supplementary Table 10**. Relative expression was calculated by the 2^−ΔΔCt method, normalized to the housekeeping gene and calibrated to the DMSO control. Melt-curve analysis confirmed single products. Each assay was run in technical triplicate with 3 independent biological replicates. Group differences were evaluated by two-sided Welch’s t-test.

#### Statistics

Unless specified, all hypothesis tests were two-sided and two-group comparisons used Wilcoxon rank-sum (bulk/scRNA-seq) or Welch’s t-test (qPCR). Correlations were assessed using Spearman’s rho for bulk-level signature/gene associations, and Pearson correlation where metacell-based ligand/receptor correlations with EZH2 were performed, with significance thresholds reported per analysis. Multiple testing was controlled using the Benjamini–Hochberg procedure, and adjusted p-values are reported as BH-adjusted P (or FDR); when unadjusted values are shown, they are explicitly labeled as nominal P.

## Results

EZH2 upregulation is progressively increased during PCa progression and associates with an immune-suppressive microenvironment.

**Figure 1:**
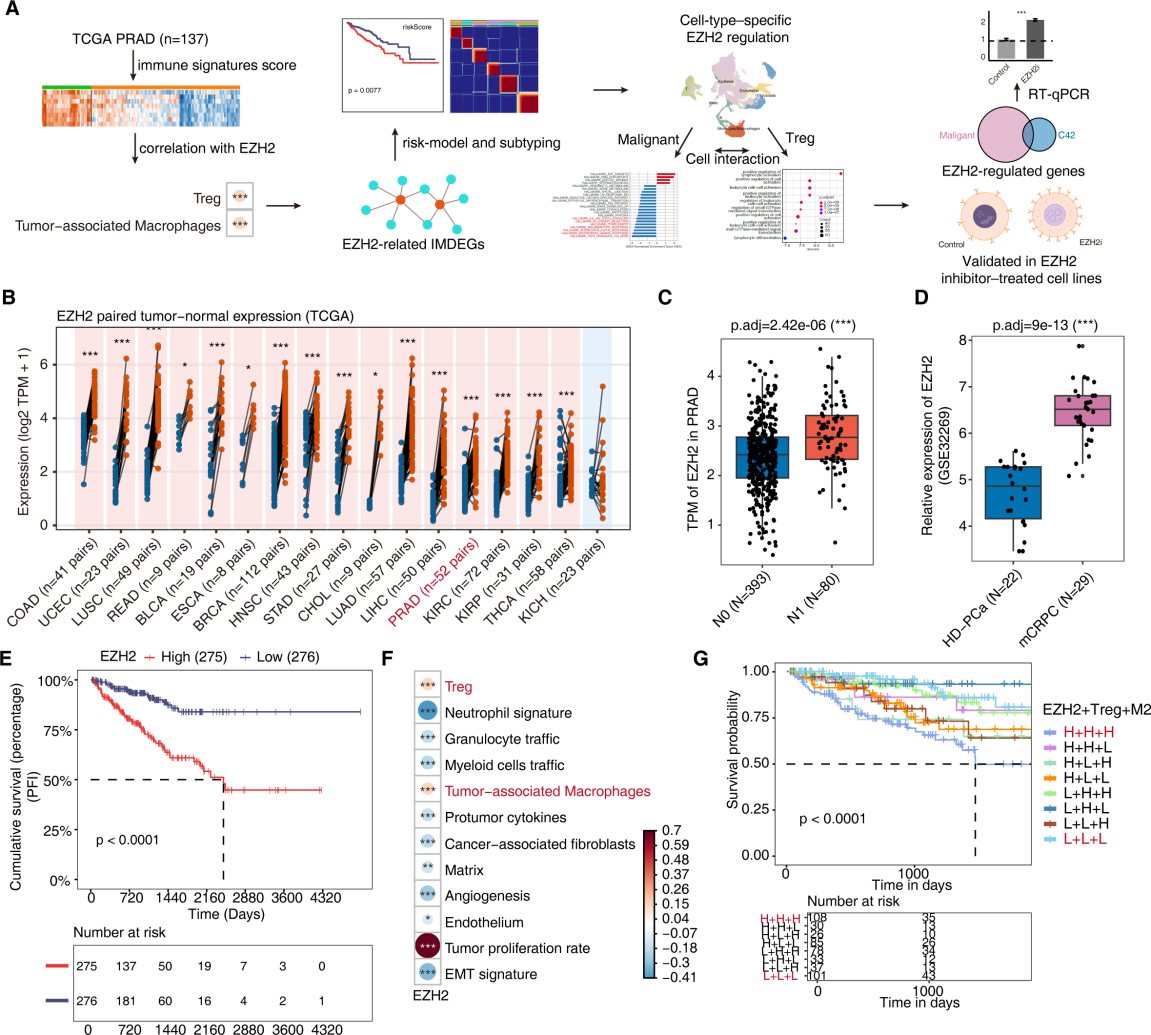
EZH2 is upregulated in PCa and associates with an immunosuppressive microenvironment and poor prognosis. **(A)** Study workflow integrating TCGA/GEO bulk datasets and single-cell analyses: immune-signature scoring, correlation with EZH2, derivation of EZH2-related IMDEGs, risk modeling and subtyping, cell-type–specific interrogation (malignant cells and Treg), cell–cell interaction profiling, and planned validation in EZH2-inhibitor–treated cell lines. **(B)** Paired tumor–normal comparison of EZH2 across TCGA cancers (Wilcoxon paired tests; *BH-adjusted P < 0.05, **BH-adjusted P < 0.01, ***BH-adjusted P < 0.001). Red and blue dots indicate tumor and matched normal samples, respectively. The red/blue panel background indicates whether EZH2 is upregulated or downregulated in tumors relative to matched normal tissues. PRAD is highlighted. **(C)** In TCGA-PRAD, EZH2 expression in lymph-node–positive tumors (N1) versus N0 tumors (Wilcoxon rank-sum test; BH-adjusted P = 2.42×10^-6^; N0: n=393, N1: n=80). **(D)** Independent GEO cohort comparing EZH2 expression between HD-PCa and mCRPC (Wilcoxon rank-sum test; BH-adjusted P = 9×10^-^¹³; HD-PCa: n=22, mCRPC: n=29). **(E)** Kaplan–Meier analysis of PFI in TCGA-PRAD comparing EZH2-high (n=275) vs. EZH2-low (n=276) tumors (log-rank test; two-sided nominal P<0.0001). **(F)** Correlations between EZH2 and immune/stromal signatures in TCGA-PRAD. Enrichment scores for 29 curated signatures were calculated by ssGSEA and z-scored within cohort; associations with EZH2 were assessed by Spearman correlation, and only signatures with nominal P < 0.05 are shown. Asterisks denote significance (*P < 0.05, **P < 0.01, ***P < 0.001). Color indicates Spearman correlation coefficient (ρ), with red denoting positive and blue denoting negative correlations. **(G)** Kaplan–Meier analysis of progression-free interval (PFI) stratified by EZH2, Treg, and TAM (M2) status in TCGA-PRAD (cutoffs as defined in Methods; log-rank test). Tick marks indicate censored observations; numbers at risk are shown below the plot. The x-axis is truncated at 5 years for clarity.

In this study, we established an integrated bulk-single-cell workflow to relate EZH2 to immune states (**Figure 1A**). In the TCGA pan-cancer survey, EZH2 expression was significantly higher in most tumors than in matched normal tissue samples in paired comparisons (**Figure 1B**) and remained elevated when unpaired tumor–normal data were analyzed (**Supplementary Figure S1A**).

Within TCGA-PRAD, EZH2 showed a progressive increase with pathological advancement (**Supplementary Figure S1B**). Cases with nodal involvement showed higher expression than node-negative disease (**Figure 1C**). An external dataset (GSE32269) generalized the gradient from hormone-dependent PCa to metastatic CRPC (**Figure 1D**). EZH2 further stratified progression-free interval (PFI), with the high-expression group relapsing earlier than the low-expression group (**Figure 1E**). Consistently, in the SU2C/PRAD 2019 cohort focusing on advanced disease, higher EZH2 was associated with inferior overall survival (**Supplementary Figure S1C**). Together, these data indicate that EZH2 is not merely a tumor–normal discriminator; its level scales with anatomic spread and therapy resistance and captures outcome risk beyond coarse staging.

Then, we performed a cohort-level correlation analysis by computing ssGSEA scores for predefined immune/stromal programs in each patient and correlating these program scores with EZH2 expression to identify tumor–microenvironment programs that co-vary with EZH2 across TCGA-PRAD samples. EZH2 showed its strongest positive association with tumor proliferation rate, and was also positively associated with Treg and TAM signature enrichment, whereas CAFs/stromal/angiogenic modules were predominantly inversely correlated with EZH2 (**Figure 1F**). These patterns are consistent with a proliferative, immune-cold state in EZH2-high tumors—one enriched for suppressive lymphoid/myeloid programs rather than effector T-cell activity (33). Prompted by this, we tested whether EZH2 synergizes with immunosuppressive signals to compound risk. Kaplan–Meier analysis of progression-free interval (PFI) stratified by the combined EZH2–Treg–TAM (M2) status revealed marked prognostic heterogeneity across the eight subgroups (log-rank *P* = 7.49 × 10^-5; **Figure 1G**). The triple-high subgroup (HHH) exhibited the poorest outcome, with a substantially reduced 5-year PFI probability (S(5y) = 0.498). In contrast, within a clinically relevant 5-year window, the EZH2^low–Treg^high–TAM^low (LHL) subgroup showed the most favorable PFI (S(5y) = 0.933; RMST at τ = 5 years = 1724 days), followed by the triple-low subgroup (LLL) (S(5y) = 0.808; RMST = 1692 days). The graded separation suggests a dose-responsive association linking EZH2 to an immunosuppressive axis that scales with clinical risk.

### EZH2-stratified IMDEGs yield a five-gene risk model and NMF-defined immune subtypes

**Figure 2:**
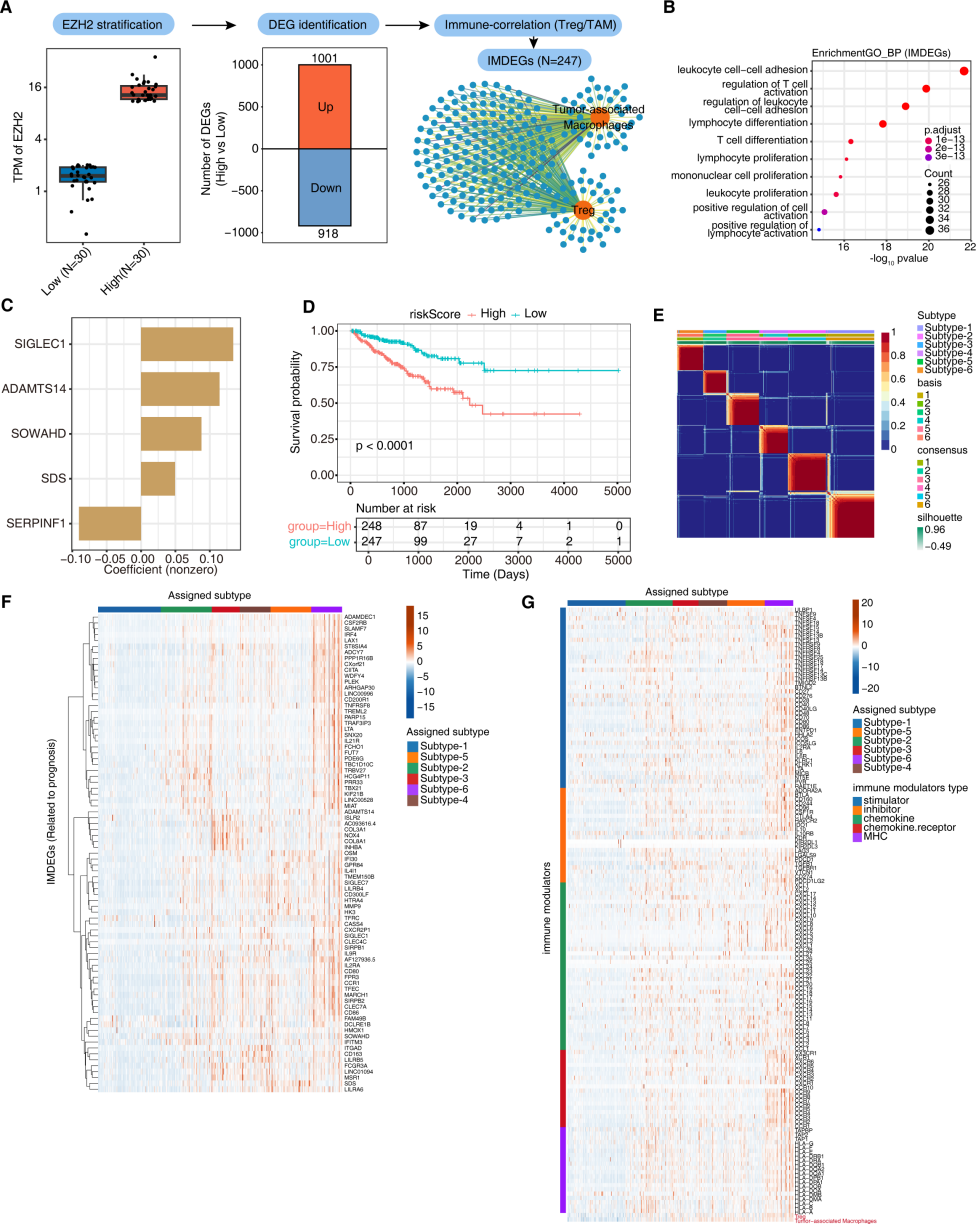
EZH2-linked IMDEGs enable risk modeling and immune subtyping of PCa. **(A)** Analysis workflow. Tumors were stratified by EZH2 (top 30 vs bottom 30; left), followed by DEG identification (center) and correlation with Treg/TAM signatures (Spearman; |r| ≥ 0.4, nominal P < 0.05) to derive IMDEGs (right; schematic network). **(B)** GO Biological Process enrichment of IMDEGs. The x-axis shows −log10(nominal P). Dot size denotes gene count; color indicates BH-adjusted P values. **(C)** Non-zero model coefficients of the final gene signature. Coefficients were obtained from the selected survival model fitted in the training cohort, and the risk score was computed as a weighted linear sum of gene expression levels using these coefficients. **(D)** Kaplan–Meier analysis of PFI in the training cohort. Patients were stratified into high- and low-risk groups based on the median risk score. The P value from the log-rank test is shown. Tick marks indicate censored observations. **(E)** Consensus Non-negative Matrix Factorization (NMF) clustering of IMDEGs with k=6. The consensus matrix shows pairwise sample co-clustering frequencies (0–1). Sample annotations indicate assigned subtype and silhouette width (range shown). Rank-selection diagnostics are provided in **Supplementary Figure S2D**. **(F)** Heatmap of prognosis-associated IMDEGs (row-scaled expression) across assigned subtypes. Rows represent individual IMDEGs and columns represent patients ordered by NMF-derived immune subtype. Expression values are row-wise z-scores, with red indicating higher-than-average and blue indicating lower-than-average expression within the cohort. **(G)** Heatmap of immune modulators across subtypes. The color scale represents row-wise z-scored expression, and column ordering is the same as in **(F)**. The left annotation indicates immune-modulator categories.

To define programs linked to EZH2, we compared the top-30 EZH2-high and bottom-30 EZH2-low TCGA-PRAD tumors (**Figure 2A**, left). Differential testing identified 1,919 DEGs (1,001 up, 918 down in EZH2-high; **Figure 2A**, middle). We then derived immune-modulated differentially expressed genes (IMDEGs) by selecting DEGs whose expression across patients was also significantly correlated with Treg or TAM signature enrichment scores, yielding 247 IMDEGs (**Figure 2A**, right; **Supplementary Table 4**). To summarize the functional themes represented by this IMDEG set, we performed Gene Ontology enrichment analysis, which highlighted terms related to T-cell activation/differentiation, lymphocyte proliferation, and cell–cell adhesion (**Figure 2B**).

To summarize EZH2-associated immune programs into clinically interpretable readouts, we used the IMDEG set as a candidate pool to derive a parsimonious prognostic signature and to stratify patients into immune subtypes. Specifically, we fitted a LASSO-penalized Cox proportional hazards model, using IMDEG expression as predictors and PFI as the survival outcome in the TCGA-PRAD training cohort. Cross-validation was used to select the optimal penalty (λ), and the resulting model retained five genes with non-zero coefficients—SIGLEC1, ADAMTS14, SOWAHD, SDS, and SERPINF1 (**Figure 2C**). Notably, SIGLEC1 (a macrophage marker) contributed the largest coefficients, consistent with a myeloid/Treg-skewed context. The resulting riskScore stratified PFI in TCGA-PRAD and remained independently prognostic after adjustment for key clinical covariates, and its prognostic separation was reproduced in SU2C/PCF using the fixed coefficients (**Figure 2D**; **Supplementary Figures S2A–C**). These results suggest that a compact, immune-informed panel distilled from EZH2 contrasts captures the transcriptomic disadvantage associated with high EZH2 and provides a practicable prognostic readout.

Unsupervised NMF of IMDEGs resolved six immune subtypes (**Figure 2E**), including a subtype marked by concurrent upregulation of immunosuppressive IMDEGs and inhibitory immune modulators (Subtype-6; **Figures 2F, G**; **Supplementary Table 5**). Together, these analyses indicate that EZH2-linked IMDEGs reflect structured tumor–immune phenotypes with prognostic relevance, providing compact signatures and subtypes that can be tested in independent cohorts and future functional studies.

### Single-cell profiling places EZH2 in malignant epithelial cells and links high EZH2 to proliferative, immune-suppressive transcriptional programs

**Figure 3:**
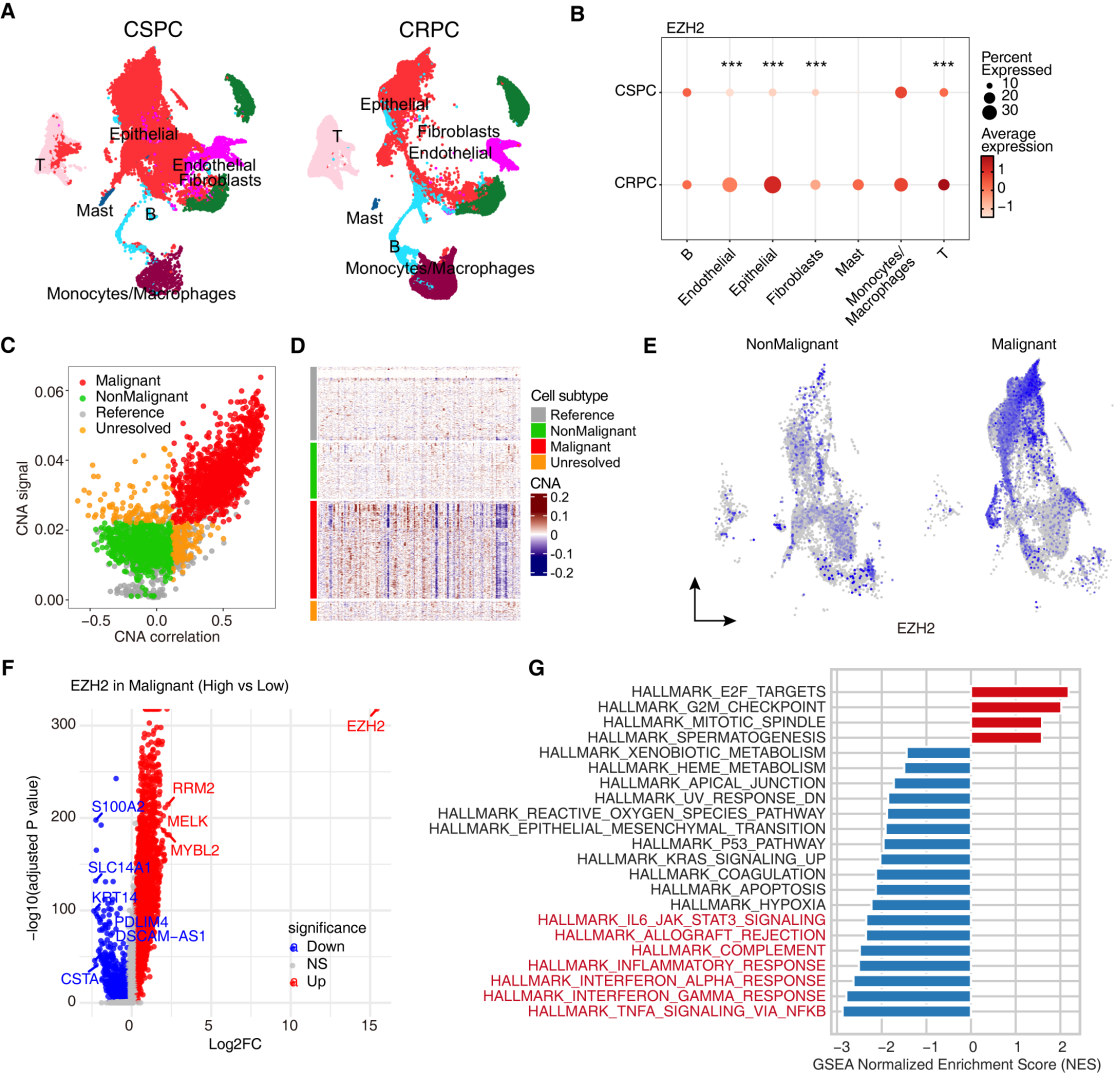
Single-cell transcriptomics identifies a malignant EZH2^high program marked by proliferation and suppression of interferon/immune-response pathways. **(A)** UMAP of scRNA-seq from CSPC and CRPC tumors with major lineages annotated. **(B)** Dot plot of EZH2 across lineages in CSPC and CRPC; color indicates average expression and dot size the percent of cells expressing, with significance denoted by asterisks (two-sided Wilcoxon rank-sum test; BH-adjusted P; ***q<0.001). **(C, D)** Malignant cell calling by copy-number inference: scatter of CNA correlation versus CNA signal separates malignant (red) from non-malignant (green) cells **(C)**; corresponding heatmap of inferred CNAs across cells **(D, E)** Feature plots of EZH2 expression distribution across malignant and non-malignant cell clusters. **(F)** Volcano plot of DEGs between malignant EZH2^high and EZH2^low cells (two-sided Wilcoxon; min.pct = 0.1, logfc.threshold = 0.25, BH-adjusted P < 0.05). **(G)** Hallmark gene set enrichment analysis (GSEA) comparing malignant EZH2^high versus EZH2^low cells; bars show normalized enrichment scores (NES), with positive NES enriched in EZH2^high and negative NES enriched in EZH2^low.

We next examined EZH2 expression and function at single-cell resolution using a scRNA-seq dataset of PCa (GSE264573). UMAP dimensionality reduction resolved the major lineages—epithelial, fibroblasts, endothelial, B/T cells, mast cells, and monocytes/macrophages—in both CSPC and CRPC samples (**Figure 3A**), and canonical markers confirmed annotations (**Supplementary Figure S3A**). Across cell types, EZH2 expression was highest in the epithelial and T cell compartment, with generally low signal in other stromal and immune lineages. EZH2 exhibits higher average expression in epithelial cells, fibroblasts, endothelial cells, and T cells of CRPC compared to CSPC (**Figure 3B**). This pattern is consistent with bulk-level observations of increased EZH2 in advanced disease.

To distinguish tumor from non-tumor epithelium, we applied copy-number–based malignancy inference. Cells classified as malignant exhibited stronger copy-number alterations (CNA) signal and lower correlation to the diploid reference, whereas non-malignant cells showed the opposite (**Figure 3C**), a pattern also evident along the genome-ordered CNA heatmap (**Figure 3D**). Feature maps further localized EZH2 to malignant clusters, with minimal expression in non-malignant cells (**Figure 3E**).

Within malignant cells, differential expression between EZH2^high and EZH2^low subsets revealed a proliferative program in the EZH2^high group, with upregulation of cell-cycle/proliferation genes (e.g., MYBL2, MELK, RRM2) and downregulation of epithelial/immune-related genes (e.g., CSTA, S100A2, SLC14A1) (**Figure 3F**; **Supplementary Table 6**). Gene set enrichment analysis (GSEA) supported these patterns, showing enrichment of E2F targets, G2M checkpoint, and mitotic spindle, alongside depletion of interferon-α/γ response, TNF-α signaling via NF-κB, complement, allograft rejection, and broader inflammatory programs (**Figure 3G**; **Supplementary Table 7**). Importantly, these malignant cell–intrinsic programs were independently reproduced in an external human CRPC scRNA-seq cohort (GSE137829, n=6 CRPC samples). In GSE137829, EZH2^high malignant cells again exhibited broad upregulation of proliferation-associated transcripts and enrichment of cell-cycle Hallmark pathways (E2F targets, G2M checkpoint, mitotic spindle), whereas inflammatory and cytokine-related programs (TNF-α signaling via NF-κB, inflammatory response, IL6–JAK–STAT3 signaling) were depleted in EZH2^high cells (**Supplementary Figures S3B, C**). Collectively, the single-cell data position EZH2 within malignant epithelial cells and associate higher EZH2 with a cell-intrinsic, cycling-dominant and immune-suppressive transcriptional state.

### Treg expansion in CRPC and EZH2-associated malignant–Treg crosstalk

**Figure 4:**
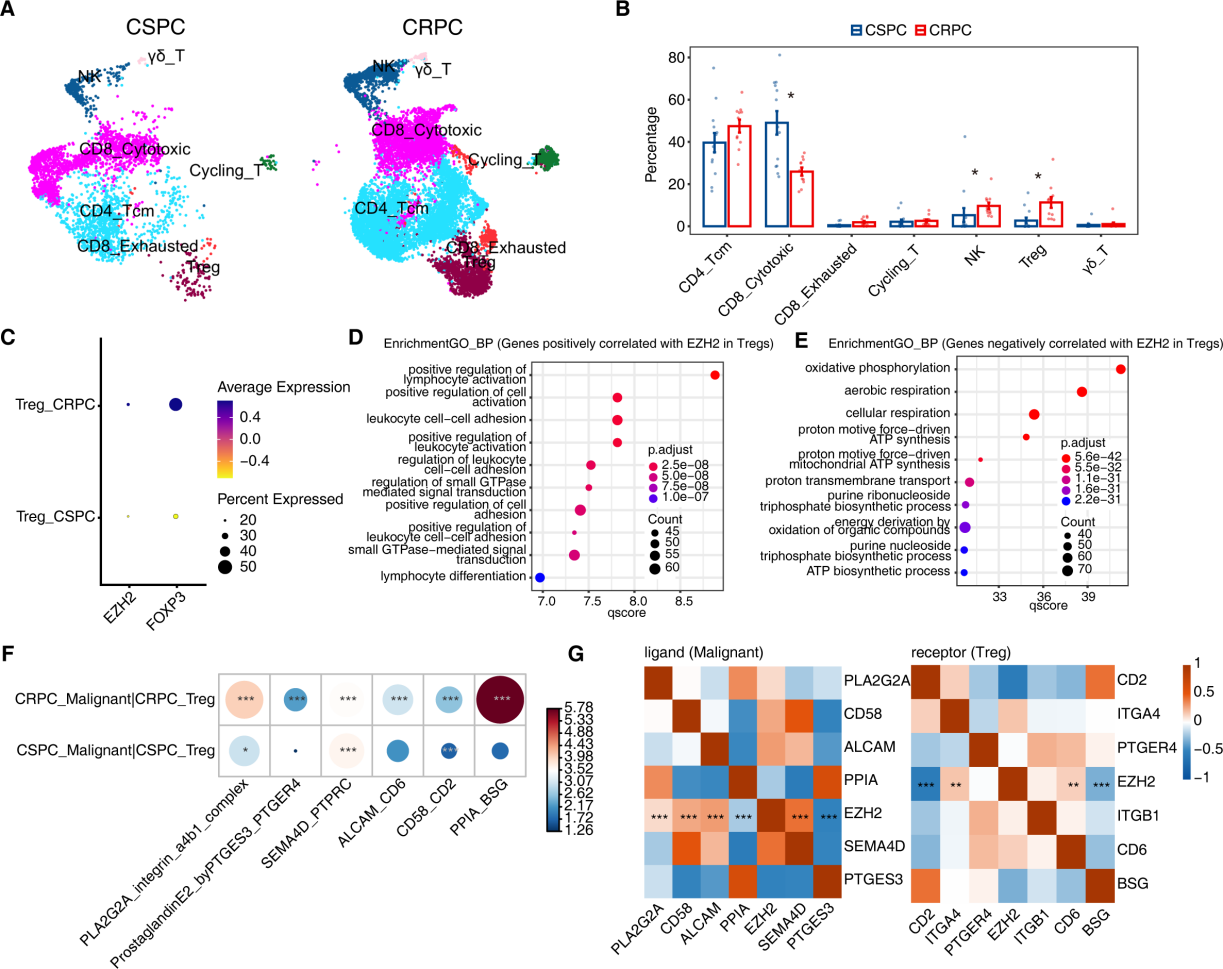
Treg expansion and EZH2-linked malignant–Treg communication in CRPC. **(A)** UMAP of T cells from CSPC and CRPC (GSE264573) with major subsets annotated. **(B)** Fractional composition of T-cell subsets. For each sample, the proportion of each T-cell subset was calculated as the number of cells assigned to that subset divided by the total number of T cells in the sample. Group differences in T-cell subtype proportions (CSPC vs CRPC) were tested using the propeller method (speckle), a limma empirical Bayes moderated t-test (two-sided) on transformed proportions, with BH FDR correction. Asterisks denote significance (* q < 0.05). **(C)** Dot plots of EZH2 and FOXP3 expression in Tregs from CSPC and CRPC samples. Dot size indicates percent expressed and color indicates average scaled expression. **(D, E)** GO Biological Process enrichment of genes positively **(D)** or negatively **(E)** correlated with EZH2 in Tregs. Correlations were assessed by metacell-based correlation analysis (see Methods; |r| ≥ 0.4, nominal P < 0.05) and GO enrichment significance is shown as BH-adjusted P (q values) in the color scale; dot size denotes gene count. **(F)** CellPhoneDB ligand–receptor analysis (mean>2; permutation P<0.05) for malignant–Treg pairs in CSPC and CRPC. Dot size and color indicate interaction mean expression level (as defined by CellPhoneDB). Significant interactions were identified by permutation testing (* P < 0.05, ** P < 0.01, *** P < 0.001). **(G)** Metacell-based correlation heatmaps linking EZH2 with malignant-cell ligands (left) and Treg receptors (right) (Pearson; ** nominal P < 0.01, *** nominal P < 0.001).

We delineated T-cell states in CSPC and CRPC samples by scRNA-seq (**Figure 4A**; CSPC: n = 3,195 T cells; CRPC: n = 10,473 T cells). Quantification of intratumoral fractions revealed that CRPC shows higher Treg and NK cells relative to CSPC, accompanied with reduced proportions of CD8^+^ cytotoxic T cells (a key effector compartment), while other T-cell states changed modestly (**Figure 4B**). This direct quantification of Treg proportions corroborates the elevated Treg signature enrichment observed in bulk RNA-seq analyses. The concurrent accumulation of Tregs and diminished proportion of CD8^+^ cytotoxic T cells is consistent with a shift toward an immune-evasive T-cell composition in therapy-resistant disease (34), whereas the NK increase likely reflects remodeling of innate lymphoid compartments rather than enhanced tumor-killing capacity.

Although higher Treg abundance in CRPC could influence interaction magnitude, **Figure 4C** shows increases in both the proportion of FOXP3^+^ Tregs and FOXP3 expression. EZH2 is mainly increased at the expression level in CRPC Tregs, supporting a Treg state shift beyond a purely compositional effect. Correlation analysis restricted to Tregs indicated that genes positively associated with EZH2 were enriched for lymphocyte activation, leukocyte cell–cell adhesion, and small GTPase–mediated signaling (**Figure 4D**; **Supplementary Table 8**), whereas negatively associated genes mapped to oxidative phosphorylation, cellular respiration, and ATP biosynthesis (**Figure 4E**; **Supplementary Table 8**). These patterns suggest that EZH2-high Tregs couple enhanced activation/adhesion programs with reduced oxidative-metabolic activity.

To further explore how EZH2-overexpressing tumor cells might interact with Tregs, we applied CellPhoneDB to malignant–Treg pairs. Several interactions displayed stronger inferred signaling in CRPC than in CSPC, including PLA2G2A–ITGA4/ITGB1 (α4β1), PTGES3–PTGER4, ALCAM–CD6, CD58–CD2, and PPIA–BSG (**Figure 4F**). Moreover, within malignant cells and Tregs respectively, the expression of key ligands (e.g., PLA2G2A, ALCAM, CD58, SEMA4D) and their cognate receptors (e.g., ITGA4, CD6) positively correlated with EZH2 levels (**Figure 4G**). Together, these associations link Treg expansion with an EZH2-connected malignant–Treg interaction network in CRPC, consistent with an immune-evasive TME, but the predicted interactions remain to be verified by functional experiments.

### EZH2 inhibition induces tumor-intrinsic transcriptional changes and reverses components of the EZH2-high malignant program in C42 cells

**Figure 5:**
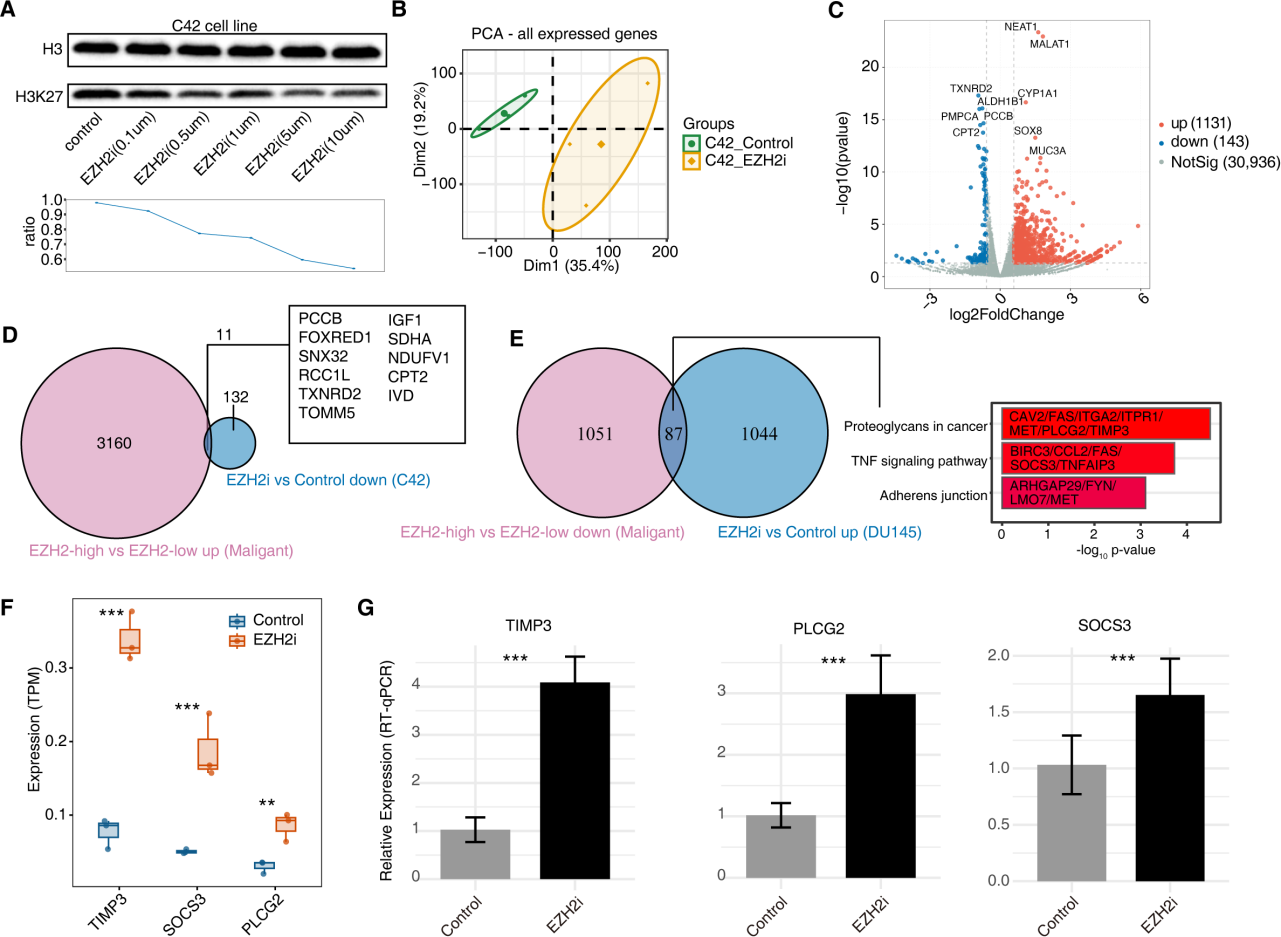
EZH2 inhibitor perturbation analysis and cross-validation with malignant-cell programs. **(A)** Immunoblot of H3K27me3 and total H3 in C42 cells treated with EZH2 inhibitor (EZH2i) at the indicated concentrations, with densitometric quantification shown as the H3K27me3/H3 ratio (line plot). **(B)** Principal component analysis (PCA) of RNA-seq expression profiles (all expressed genes) comparing C42_Control and C42_EZH2i groups. **(C)** Volcano plot of differentially expressed genes (DEGs) between C42_EZH2i and C42_Control. Upregulated genes are shown in red, downregulated genes in blue, and non-significant genes in gray (two-sided Wald test in DESeq2; BH-adjusted P < 0.05 and |log2FC| ≥ 0.585). The top 5 upregulated (NEAT1, MALAT1, CYP1A1, SOX8, MUC3A) and top 5 downregulated (TXNRD2, ALDH1B1, PMPCA, PCCB, CPT2) genes are labeled (ranked by BH-adjusted P among significant DEGs). **(D)** Venn diagram showing overlap between malignant-cell DEGs from single-cell analysis (EZH2^high vs EZH2^low) and genes downregulated by EZH2i in C42 cells; representative overlapping genes are listed. **(E)** Venn diagram showing overlap between genes downregulated in EZH2^high malignant cells and genes upregulated by EZH2i in C42 cells; top3 enriched KEGG pathways for the overlapping set are shown on the right. **(F)** RNA-seq expression (TPM) of selected genes in Control versus EZH2i conditions. Box plots show median and IQR; points represent biological replicates. Differential expression was assessed using the two-sided Wald test in DESeq2 with BH-adjusted P values; significance is indicated as **q < 0.01 and ***q < 0.001. **(G)** RT–qPCR validation of selected genes in Control versus EZH2i conditions. Data are shown as mean ± SD (n = 3 biological replicates); between-group comparisons were performed using two-sided Student’s t-test; ***P < 0.001. GAPDH was used as the internal control and relative expression was calculated using the 2^-ΔΔCt method.

Using the CRPC model cell line C42, we evaluated the pharmacodynamic and transcriptomic consequences of EZH2 inhibition. Immunoblotting demonstrated a progressive decrease in H3K27me3 with increasing doses of tazemetostat, a selective EZH2 inhibitor, and densitometric quantification (H3K27me3 normalized to total H3) showed a corresponding monotonic decline in the H3K27me3/H3 ratio, supporting dose-dependent on-target inhibition of EZH2 catalytic activity (**Figure 5A**). At the transcriptome level, PCA based on global gene expression profiles revealed a clear separation between C42_Control and C42_EZH2i samples, indicating that EZH2 blockade drives a broad transcriptional reprogramming in a tumor-intrinsic setting (**Figure 5B**). Differential expression analysis identified a substantial set of EZH2i-responsive genes, with an overall bias toward gene induction, consistent with the canonical role of EZH2/PRC2 as a transcriptional repressor whose inhibition can derepress silenced programs (**Figure 5C**). In the volcano plot (**Figure 5C**), we labeled the top 5 upregulated (NEAT1, MALAT1, CYP1A1, SOX8, MUC3A) and top 5 downregulated (TXNRD2, ALDH1B1, PMPCA, PCCB, CPT2) (ranked by BH-adjusted P among significant DEGs), highlighting representative EZH2i-responsive transcriptional changes in C42 cells.

To link the perturbation response to the malignant-cell states inferred from scRNA-seq, we cross-compared tazemetostat-induced DEGs in C42 cells with malignant-cell programs defined by EZH2-high versus EZH2-low stratification. Overlap analysis demonstrated 11 overlapping genes between the upregulated genes in EZH2-high malignant cells and the downregulated genes in the EZH2i group; additionally, there were 87 overlapping genes between the suppressed genes in EZH2-high malignant cells and the upregulated genes in the EZH2i group, including PLCG2 and TIMP3 involved in the “proteoglycans in cancer”, as well as SOCS3 involved in the “TNF signaling pathway” (**Figures 5D, E**; **Supplementary Table 9**). Collectively, these concordant directional changes provide support that the EZH2^high malignant state captured by scRNA-seq reflects a pharmacologically modulated tumor-intrinsic transcriptional program. Both RNA-seq and reverse RT-qPCR validated that EZH2i significantly upregulated the expression of TIMP3, PLCG2, and SOCS3 (**Figures 5F, G**). It is well established that proteoglycans promote tumor cell proliferation by regulating extracellular matrix remodeling and activation of proliferative signaling pathways (35), while the TNF signaling pathway participates in tumor immune suppression by modulating inflammatory responses and immune cell functions (36). Together, EZH2 inhibition in C42 cells reduced H3K27me3 and shifted a tumor-intrinsic transcriptional program in the opposite direction of the EZH2-high malignant signature, including induction of TIMP3/PLCG2/SOCS3, supporting these genes as tractable downstream nodes for follow-up mechanistic and combination-therapy studies.

## Discussion

Here we delineate how EZH2-high states in advanced PCa align with an immunosuppressive context across patient-level cohorts and cell-type–resolved analyses. Across cohorts, EZH2-high tumors associate with worse outcomes and Treg/TAM-skewed programs; at single-cell resolution, these signals localize to EZH2-high malignant epithelial programs and increased Treg representation in CRPC; *in vitro* EZH2 inhibition provides orthogonal support for a tumor-intrinsic transcriptional program consistent with these patient-derived associations.

Bulk analyses across TCGA and external cohorts supported that EZH2 increases with disease advancement and tracks adverse prognosis, consistent with prior work connecting EZH2 to aggressive PCa biology (37). From a microenvironment perspective, EZH2-high tumors showed higher enrichment of Treg- and TAM/M2-related signatures and poorer outcomes in the EZH2-high/Treg-high/TAM-high phenotype group, aligning with evidence that EZH2 contributes to immunosuppressive circuits involving Tregs and macrophage-associated programs (38). Notably, associations varied across stromal/angiogenic signatures, indicating a program-specific immune context rather than a uniform shift of all non-malignant components.

To move beyond single-signature correlations, we derived IMDEGs that capture transcriptional programs at the intersection of EZH2 stratification and Treg/TAM-associated signatures. IMDEG-based modeling and NMF subtyping summarized co-varying tumor–immune states and highlighted a subtype characterized by broad upregulation of immunosuppressive mediators and inhibitory checkpoints, paralleling the EZH2-high immunosuppressive context observed at the cohort level. Among the model genes, SERPINF1 has been implicated in tumor immune microenvironment remodeling and immune escape in prior studies, inducing through associations with macrophage programs and stemness-related pathways (39). Rather than over-interpreting individual genes, these IMDEG-based readouts provide a compact and clinically interpretable representation of EZH2-associated immune states that can be tested in future cohorts and functional systems.

Single-cell analyses complemented the bulk findings by pinpointing EZH2 expression and associated programs within specific compartments. CNA-based inference identified malignant epithelial populations with the highest EZH2 expression. Moreover, EZH2 expression in CRPC malignant cells was significantly higher than that in CSPC cells. In malignant cells with high EZH2 expression, proliferation-associated genes were upregulated, and GSEA shows significant activation of cell cycle-related pathways, which is consistent with previous studies reporting that EZH2 promotes PCa proliferation (40, 41). Concurrently, EZH2-high malignant cells showed reduced expression of immune-response programs, consistent with PRC2-linked repression of epithelial/immune-associated genes and impaired antigen-presentation–related signals (42). Together, these results support a co-occurring “high proliferation/low immunogenicity” malignant state associated with elevated EZH2.

In parallel, CRPC tumors showed higher intratumoral Treg proportions and elevated EZH2 in tumor-infiltrating Tregs, consistent with evidence that Tregs accumulate in advanced PCa and sustain suppressive phenotypes in the TME (43, 44). Using CellPhoneDB, we observed stronger predicted malignant–Treg interaction signals in CRPC and positive associations with EZH2 expression; these interactions should be viewed as hypothesis-generating candidates rather than confirmed signaling, with representative axes such as PLA2G2A–integrin α4β1 and ALCAM–CD6 supported by prior immunoregulatory literature (45). These results nominate testable molecular bridges linking EZH2-high tumor states to Treg-associated immunosuppression in CRPC.

Pharmacologic EZH2 inhibition in the CRPC-relevant C42 cell line reduced H3K27me3, supporting on-target activity, and induced coordinated transcriptional changes including upregulation of TIMP3, PLCG2, and SOCS3 validated by RT–qPCR. These directions are consistent with prior evidence that EZH2 can epigenetically repress tumor-suppressive programs such as TIMP3 in PCa (46), and with reported links between PLCG2/SOCS3 and immune-relevant and cytokine/STAT-related signaling (60, 61, 62). Given the absence of immune cells in this perturbation setting and the use of a single cell line, these data provide a tractable, on-target perturbational link between EZH2 activity and tumor transcriptional programs, nominating TIMP3/PLCG2/SOCS3 as testable downstream nodes for future mechanistic and combination-therapy studies.

Collectively, our multi-layer analysis adds an integrated view of EZH2 in advanced PCa by linking cohort-level immunosuppressive programs to cell-type–resolved malignant and Treg states, nominating testable malignant–Treg interaction candidates, and providing perturbational transcriptional support in a CRPC-relevant model. However, several limitations should be acknowledged. First, our bulk and single-cell analyses are largely associative, and we lack *in vivo* evidence to establish a causal role of EZH2 in shaping Treg/TAM-related immunosuppressive features and immune evasion. Second, the ligand–receptor results are based on expression-driven inference, and functional validation of the highlighted interaction axes, as well as direct regulatory links between EZH2 and downstream genes, remains limited. Third, our perturbation experiments were performed in a single CRPC-relevant cell line (C42) without immune context, which constrains mechanistic interpretation and translational generalization. Finally, regarding cohort representativeness, the single-cell analyses rely primarily on GSE264573, and additional multi-center, larger CRPC scRNA-seq cohorts are needed to confirm the stability and generalizability of EZH2-associated phenotypes. Future work will therefore prioritize (i) *in vivo*/xenograft or co-culture models to test causality between EZH2 perturbation and tumor–immune phenotypes, (ii) genetic perturbation and chromatin assays (e.g., knockout/overexpression and ChIP-seq) to establish direct regulatory targets and validate key interaction axes, (iii) expansion to multi-center CRPC single-cell cohorts for external validation, and (iv) evaluation of EZH2 inhibitors in rational combination with immune checkpoint blockade or other targeted therapies in preclinical settings.

## Data Availability

The datasets presented in this study can be found in online repositories. The names of the repository/repositories and accession number(s) can be found in the article/**Supplementary Material**.
