# Supplementary material for: Integrative bulk and single-cell transcriptomics link EZH2 to immunosuppressive programs and tumor–Treg crosstalk in castration-resistant prostate cancer
Source: Front Immunol. 2026 Feb 3;17:1725097. doi: 10.3389/fimmu.2026.1725097 (PMC12909565; doi:10.3389/fimmu.2026.1725097)
Supplement: Supplementary file 1 [file DataSheet1.zip › Supplementary Figures.DOCX]

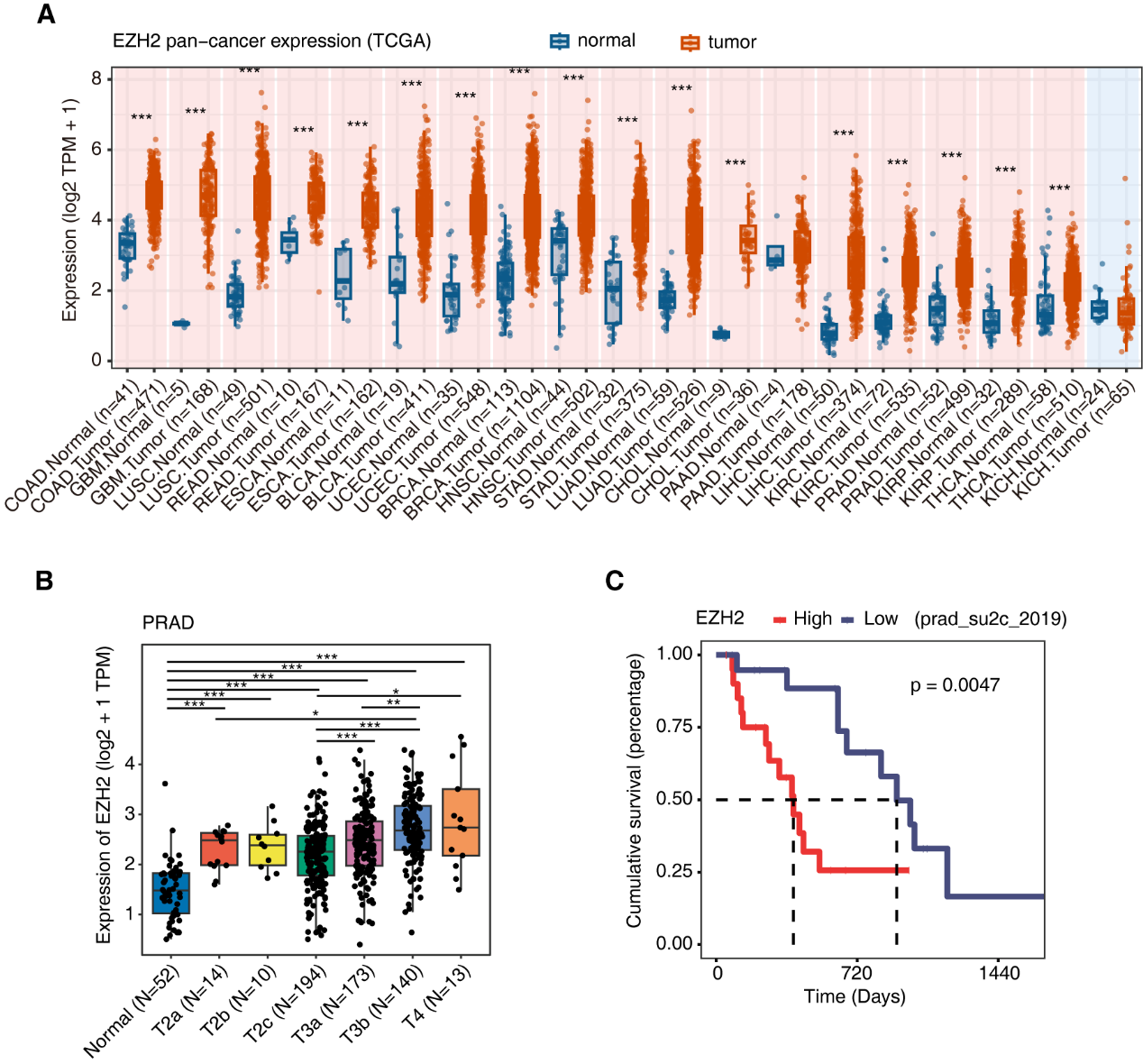


**Supplementary Fig. S1. Pan-cancer elevation of EZH2, stage-wise increase in PRAD, and validation of prognostic value in an external cohort.**
(A) Unpaired tumor–normal comparison of EZH2 across TCGA cancer types. Boxplots show log2(TPM + 1) expression; dots indicate individual samples; n’s are shown on the x-axis labels. Two-sided Wilcoxon rank-sum tests with Benjamini–Hochberg correction were used; significance is denoted as * FDR < 0.05, ** FDR < 0.01, *** FDR < 0.001.

(B) Stepwise increase of EZH2 within TCGA-PRAD across pathological stages (T2a–T4). Overall differences were assessed by Kruskal–Wallis.

(C) External validation of prognosis in the prad_su2c_2019 cohort. Patients were dichotomized by the median EZH2 expression, and overall survival was compared by a two-sided log-rank test (p = 0.0047).

c
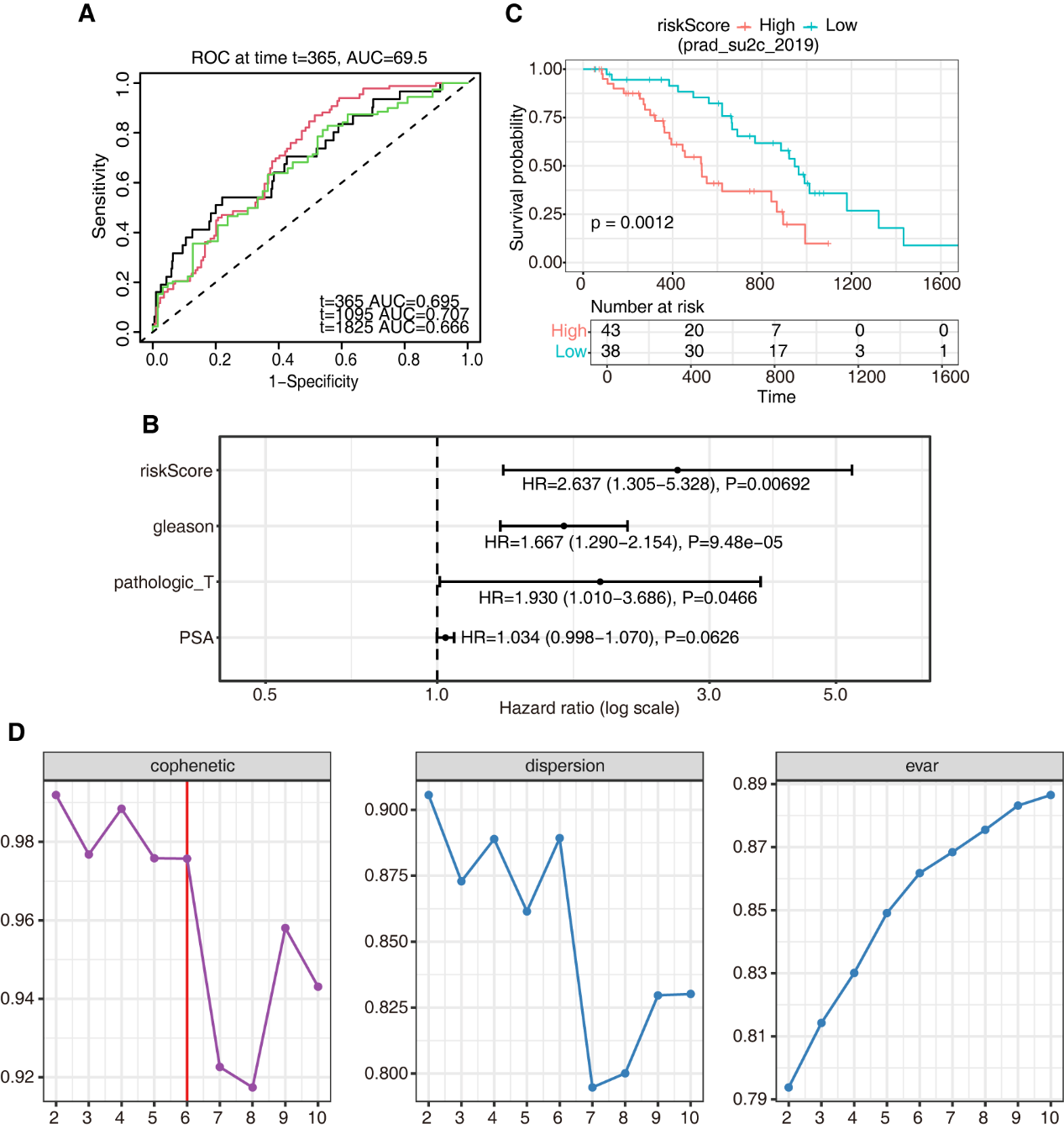


**Supplementary Fig. S2. External validation of the four-gene IMDEG risk model and determination of the subtype number.**

(A) Time-dependent ROC curves for the selected model in the training cohort at pre-specified time points (1, 3, and 5 years), with corresponding AUC values annotated.

(B) Multivariable Cox proportional hazards analysis including the risk score and clinical covariates (Gleason score, pathological T stage, and PSA). Forest plot shows hazard ratios (HRs) with 95% confidence intervals (CIs) and Wald test P values.

(C) External validation in the SU2C/PCF 2019 cohort (prad_su2c_2019). Kaplan–Meier OS curves comparing high- vs low-risk groups stratified by the median risk score, where the risk score was calculated using the coefficients learned in the training cohort without re-fitting. The log-rank test P value is shown. Tick marks indicate censored observations.

(D) Choice of cluster/rank for IMDEG subtyping. Diagnostic curves (cophenetic correlation, dispersion, and average silhouette width; k = 2–10) and the k-means elbow/silhouette profiles indicate k = 6 as the optimal solution.


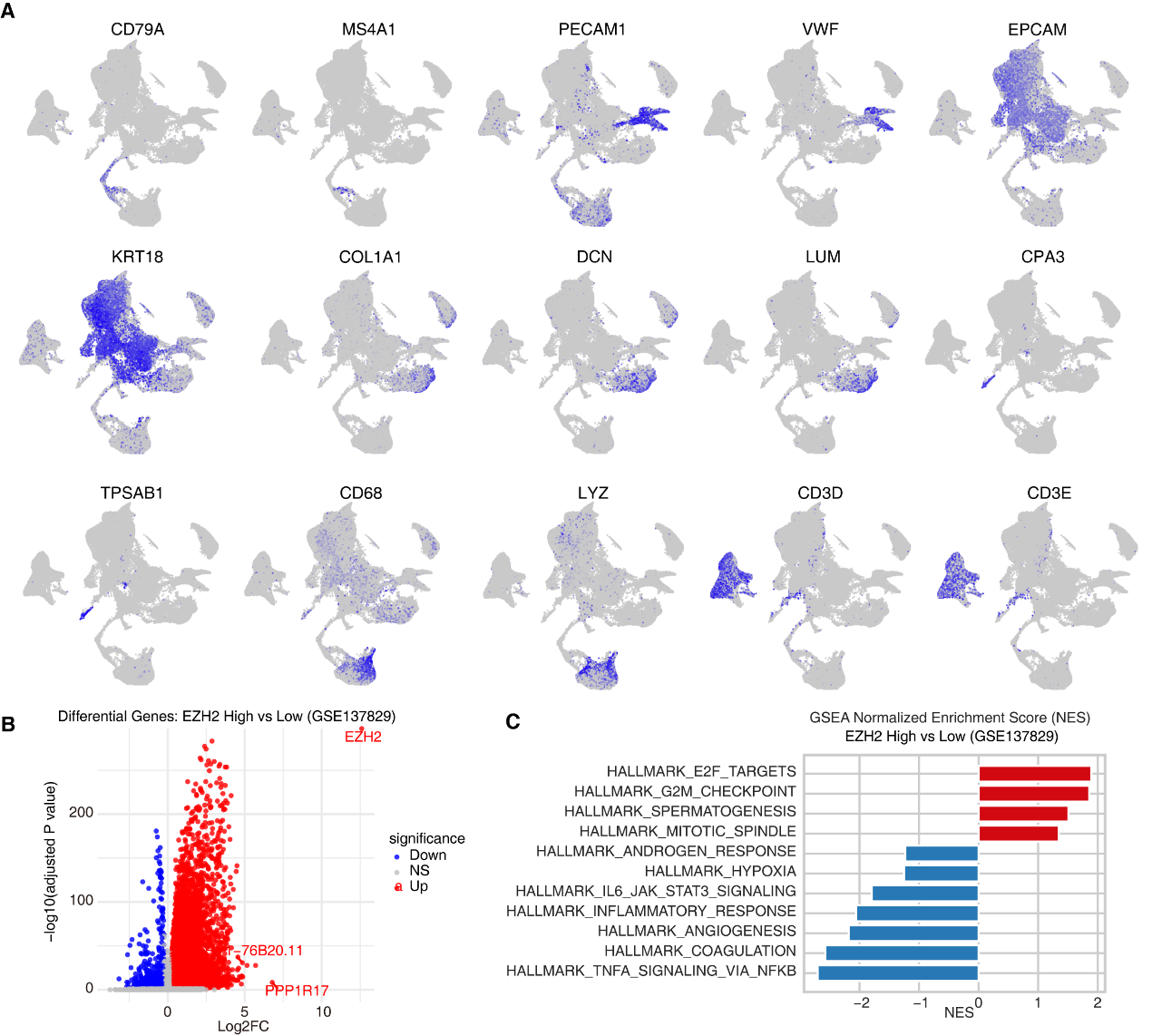


**Supplementary Fig. S3. Canonical marker validation of cell-type annotations in the prostate scRNA-seq dataset.**

(A) Feature plots of representative lineage markers on the same UMAP embedding as in Fig. 3A. Markers include B cells (CD79A, MS4A1), Endothelial cells (PECAM1, VWF), Epithelial cells (EPCAM, KRT18), Fibroblasts (COL1A1, DCN, LUM), Mast cells (CPA3, TPSAB1), Monocytes/Macrophages (CD68, LYZ), and T cells (CD3D, CD3E). Blue intensity reflects higher normalized expression in individual cells; grey dots indicate other cells.

(B) Differential gene expression between EZH2^high and EZH2^low malignant cells in the independent cohort GSE137829. Volcano plot showing differentially expressed genes comparing EZH2^high versus EZH2^low malignant cells. The x-axis indicates log2 fold change and the y-axis indicates −log10(adjusted P value).

(C) GSEA of Hallmark pathways for EZH2^high versus EZH2^low malignant cells in GSE137829. Bar plot of normalized enrichment scores (NES) from GSEA using Hallmark gene sets based on the preranked differential expression results. Positive NES (red) indicates pathways enriched in EZH2^high cells, and negative NES (blue) indicates pathways enriched in EZH2^low cells. Pathways are ordered by NES.
